# Phenotypically concordant distribution of pick bodies in aphasic *versus* behavioral dementias

**DOI:** 10.1186/s40478-024-01738-7

**Published:** 2024-02-22

**Authors:** Allegra Kawles, Rachel Keszycki, Grace Minogue, Antonia Zouridakis, Ivan Ayala, Nathan Gill, Alyssa Macomber, Vivienne Lubbat, Christina Coventry, Emily Rogalski, Sandra Weintraub, Qinwen Mao, Margaret E. Flanagan, Hui Zhang, Rudolph Castellani, Eileen H. Bigio, M.-Marsel Mesulam, Changiz Geula, Tamar Gefen

**Affiliations:** 1 Mesulam Center for Cognitive Neurology and Alzheimer’s Disease, Northwestern University Feinberg School of Medicine, Chicago, IL USA; 2 Department of Psychiatry & Behavioral Sciences, Northwestern University Feinberg School of Medicine, Chicago, IL USA; 3 Department of Preventive Medicine, Northwestern University Feinberg School of Medicine, Chicago, IL USA; 4 Department of Neurology, University of Chicago School of Medicine, Chicago, IL USA; 5 Department of Pathology, Northwestern University Feinberg School of Medicine, Chicago, IL USA; 6 Department of Neurology, Northwestern University Feinberg School of Medicine, Chicago, IL USA; 7 Department of Cell and Developmental Biology, Northwestern University Feinberg School of Medicine, Chicago, USA

**Keywords:** Pick’s disease, Stereology, Primary progressive aphasia, Behavioral variant frontotemporal dementia, Frontotemporal lobar degeneration, Tau

## Abstract

Pick’s disease (PiD) is a subtype of the tauopathy form of frontotemporal lobar degeneration (FTLD-tau) characterized by intraneuronal 3R-tau inclusions. PiD can underly various dementia syndromes, including primary progressive aphasia (PPA), characterized by an isolated and progressive impairment of language and left-predominant atrophy, and behavioral variant frontotemporal dementia (bvFTD), characterized by progressive dysfunction in personality and bilateral frontotemporal atrophy. In this study, we investigated the neocortical and hippocampal distributions of Pick bodies in bvFTD and PPA to establish clinicopathologic concordance between PiD and the salience of the aphasic *versus* behavioral phenotype. Eighteen right-handed cases with PiD as the primary pathologic diagnosis were identified from the Northwestern University Alzheimer’s Disease Research Center brain bank (bvFTD, N = 9; PPA, N = 9). Paraffin-embedded sections were stained immunohistochemically with AT8 to visualize Pick bodies, and unbiased stereological analysis was performed in up to six regions bilaterally [middle frontal gyrus (MFG), superior temporal gyrus (STG), inferior parietal lobule (IPL), anterior temporal lobe (ATL), dentate gyrus (DG) and CA1 of the hippocampus], and unilateral occipital cortex (OCC). In bvFTD, peak neocortical densities of Pick bodies were in the MFG, while the ATL was the most affected in PPA. Both the IPL and STG had greater leftward pathology in PPA, with the latter reaching significance (*p* < 0.01). In bvFTD, Pick body densities were significantly right-asymmetric in the STG (*p* < 0.05). Hippocampal burden was not clinicopathologically concordant, as both bvFTD and PPA cases demonstrated significant hippocampal pathology compared to neocortical densities (*p* < 0.0001). Inclusion-to-neuron analyses in a subset of PPA cases confirmed that neurons in the DG are disproportionately burdened with inclusions compared to neocortical areas. Overall, stereological quantitation suggests that the distribution of neocortical Pick body pathology is concordant with salient clinical features unique to PPA vs. bvFTD while raising intriguing questions about the selective vulnerability of the hippocampus to 3R-tauopathies.

**Supplementary Information:**

The online version contains supplementary material available at 10.1186/s40478-024-01738-7.

## Introduction

Frontotemporal lobar degeneration with tauopathy (FTLD-tau) is a neurodegenerative disease found at autopsy and is the second most common cause of dementia under age 65 [53]. Much of the complexity in studying FTLD-related dementias is due in part to the phenomenon that the same pathology can be associated with different clinical dementia syndromes. For example, primary progressive aphasia (PPA) is a syndrome characterized by isolated and progressive impairment of language and focal atrophy of regions in the language dominant hemisphere [30, 33, 34, 51]. In contrast, behavioral variant frontotemporal dementia (bvFTD) is characterized by progressive dysfunction in personality and atrophy in bilateral frontal regions [1, 28, 48, 54]. Yet, both distinct clinical phenotypes can be associated with the same underlying pathology. In this study, we focus on PPA and bvFTD caused by the FTLD-tau subtype known as Pick’s disease [48].

Pick’s disease (PiD) is one of three major subtypes of FTLD-tau, first coined by Arnold Pick who observed patients with progressive behavior and/or language deficits and focal frontotemporal atrophy [24]. Those affected with PiD commonly display “knife-edge” frontal lobe as well as anterior temporal lobe atrophy on structural MRI and subsequently at autopsy [45, 62]. It is now understood that PiD is defined by the postmortem neuropathology characterized by distinct, round cytoplasmic neuronal tau inclusions called Pick bodies [21, 57]. PiD is considered a 3R-tauopathy, as its pathologic inclusions consist almost exclusively of pathologic tau containing three microtubule-binding repeat domains [11]. Conversely, corticobasal degeneration (CBD) and progressive supranuclear palsy (PSP) are 4R-tauopathies, while neurofibrillary tangles in Alzheimer’s disease (AD) consist of both 3R and 4R tau [7, 12, 16].

The goal of the present study was to investigate the neocortical and hippocampal distributions of Pick bodies in PPA and bvFTD to establish clinicopathologic concordance between PiD and the salience of the aphasic versus behavioral phenotype. Utilizing immunohistochemical techniques and an unbiased stereologic approach, we analyzed up to seven regions, six of which were acquired bilaterally, to closely investigate the relationship between anatomy, clinical syndrome, and regional and hemispheric distributions of Pick bodies. Findings provide further evidence of the notion that dementia symptoms are related to localization of pathology, while also presenting evidence of 3R tau selective vulnerability.

## Materials and methods

### Case characteristics

Eighteen right-handed cases with autopsy-confirmed PiD as the primary pathologic diagnosis were identified from the NIA-funded Alzheimer’s Disease Research Center Brain Bank housed within the Mesulam Center for Cognitive Neurology and Alzheimer’s Disease at Northwestern University’s Feinberg School of Medicine. Written informed consent was obtained from all participants who committed to brain donation. Nine cases carried an antemortem diagnosis of bvFTD and nine carried an antemortem diagnosis of PPA, and half the total cohort was female. All cases with a clinical diagnosis of PPA were co-enrolled in the Northwestern PPA program. In the bvFTD cohort, the mean age at symptom onset was 58.44 years (SD = 7.86), and the mean age at death was 69.11 years (SD = 7.52). The mean age at onset for the PPA cohort was 59.44 years (SD = 4.75), and the mean age at death was 69.44 years (SD = 4.1).

Across the total cohort, disease duration ranged from 4 to 14 years (M = 10.33 years, SD = 3.14). The mean PMI was 17.5 h, and the mean brain weight was 1008.25 g. A diagnosis of bvFTD was based on the 2011 criteria of the International Behavioral Variant FTD Consortium [48]. The diagnosis of PPA was based on the criteria of Mesulam [32] and required a clinical history of progressive language impairment unaccompanied by consequential decline in other cognitive domains within the initial stages of the disease [29, 35]. Further classification into PPA subtypes was based on retrospective chart review by a neurologist (MMM) guided by the criteria of Gorno-Tempini et al. [18] and Mesulam et al. [31]. Of the PPA cases, 6 were clinically diagnosed with the agrammatic variant and one with the semantic variant; one case was too severe at enrollment to classify, and another demonstrated a “mixed” phenotype, defined by deficits in both grammar and verbal semantics [31]. One PPA case (Case 14) had a substantially reduced disease duration due to a non-dementia-related death. At her last and final clinical visit, her overall dementia severity was still very mild (global clinical dementia rating (CDR) = 0.5), and language was mildly impaired (CDR language = 1). Fifteen cases had genotyping for apolipoprotein E (ApoE). Four cases had an ApoE ε4 allele, the strongest known genetic risk factor for amnestic Alzheimer’s dementia associated with AD [3, 8, 13] but not with PPA associated with AD [52, 58], and two cases had an ApoE ε2 allele, a proposed protective allele against AD [6, 49]. No cases had a known mutation associated with AD or FTLD. See Table 1 for individual case characteristics.

**Table 1:** Case characteristics

Case	Sex	PMI (h)	Age at Death (Years)	Disease Duration (Years)	Brain weight (g)	Education (years)	Clinical Dx	PPA Subtype	ApoE	Comorbid pathologic diagnoses
1	F	25	78	12	1010	20	bvFTD	–	3,4	Moderate vascular disease, Low ADNC (A0,B1,C1)
2	M	21	67	8	990	20	bvFTD	–	2,3	Vascular disease
3	F	2	71	14	661	18	bvFTD	–	3,3	Mild vascular disease
4	F	13	58	12	866	12	bvFTD	–	2,3	Low ADNC (A0,B1,C0), Fahr’s
5	F	9	82	12	890	18	bvFTD	–	3,3	Low ADNC (A2,B1,C2), moderate vascular disease
6	M	24	70	11	1105	12	bvFTD	–	3,3	Low ADNC (A1,B1,C2), moderate vascular disease
7	M	7	66	6	988	14	bvFTD	–	UNK	Low ADNC (A1,B0,C2), mild vascular disease
8	M	24	61	14	880*	18	bvFTD	–	UNK	Unilateral hippocampal sclerosis, moderate vascular disease
9	M	49	69	7	1133	16	bvFTD	–	3,3	Low ADNC (A2,B0,C1), mild to moderate vascular disease
10	M	5	70	14	1018	20	PPA	G	3,3	Mild vascular disease, Low ADNC (A0,B1,C0)
11	F	28	64	11	990	16	PPA	S	3,4	Hippocampal sclerosis, moderate vascular disease
12	M	UNK	65	12	1055	14	PPA	G	3,3	Moderate vascular disease
13	M	27	75	9	1125	14	PPA	Other – Mixed	UNK	Low ADNC (A3,B1,C3), mild atherosclerosis
14	F	UNK	67	4	1140*	12	PPA	G	3,4	Low ADNC (A2,B1,C3), 2 acute parenchymal hematomas, moderate vascular disease
15	M	5	69	7	1100	12	PPA	Other – Severe	3,3	Moderate vascular disease, left temporal pole subacute infarct, low ADNC (A1,B0,C1)
16	F	3.5	76	12	965	16	PPA	G	3,4	Moderate vascular disease
17	F	14	68	8	1010	16	PPA	G	3,3	Mild vascular disease, low ADNC (A3,B0,C2)
18	F	24	71	14	1226	17	PPA	G	3,3	Moderate vascular disease

*bvFTD* Behavioral variant frontotemporal dementia, *PPA* Primary progressive aphasia, *M* Male, *F* Female, *PMI* Postmortem interval, *UNK* Unknown, *Fixed, *ApoE* Apolipoprotein E, *G* Agrammatic, *S* Semantic, *ADNC* Alzheimer’s disease neuropathologic change

### Neuropathologic evaluation and histological preparation

Following autopsy, the cerebral hemispheres were separated in the midsagittal plane, cut into 2- to 3-cm coronal slabs, fixed in formalin or 4% paraformaldehyde for 36 h, taken through sucrose gradients (10%–40%) for cryoprotection, and stored in 40% sucrose with 0.02% sodium azide at 4° C. Gross examination after autopsy showed severe atrophy in frontotemporal regions across all cases, with more severe parietal atrophy in PPA cases. The pathologic diagnosis of FTLD and specification of its variants was rendered by neuropathologists (EHB, QM, RJC, and MEF) using published consensus criteria of the Consortium for FTLD [4]. No cases showed greater than “low” Alzheimer’s Disease neuropathologic change (ADNC) according to criteria set by Hyman et. al (2012) and Montine et. al (2012) [20, 44], and those with co-morbid ADNC showed minimal neurofibrillary tangle pathology (i.e., Braak stage 0 or 1). Furthermore, all cases showed Pick’s disease as the primary pathologic diagnosis, with no lesions of other tauopathies observed. For all cases, samples were taken from 5 regions bilaterally [middle frontal gyrus (MFG; BA 8–9), superior temporal gyrus (STG; BA 22), inferior parietal lobule (IPL; BA 39–40), and dentate gyrus (DG) and CA1 regions of the posterior third of hippocampus complex] and unilateral left occipital cortex (OCC; BA 17). In PPA cases, additional bilateral samples were taken from the anterior temporal lobe (ATL; BA 38) as a part of standardized neuropathologic protocol for the Northwestern University PPA Program. Regions of interest were embedded in paraffin and cut into 5 μm-thick sections. Sections were stained immunohistochemically with the AT8 antibody, which recognizes human tau phosphorylated at Ser202/205 (mouse monoclonal; Invitrogen MN1020; 1/500), using the Avidin–Biotin Complex (ABC)-based method with streptavidin to visualize Pick bodies. Slides were developed manually using either amino ethyl carbazol (AEC) chromogen (NovaRed; Vector Laboratories SK4800), which gave tau pathology a red hue, or automatically using 3,3’-Diaminobenzidine (DAB) chromogen in a Leica Bond-Max Autostainer, which gave pathology a brown hue. There was no qualitative difference in pathologic tau immunopositivity and staining between chromogens or methods, and control slides were used during manual staining to ensure comparable immunostaining between batches. A subset of PPA cases (Cases 10, 16, and 17) was histologically stained using 1.0% cresyl violet to visualize neurons for counting.

### Modified stereological quantitative analysis of Pick pathology

Modified stereological analysis was carried out on five adjacent sections employing the fractionator method and the StereoInvestigator software (MicroBrightField) to estimate the density of Pick bodies and, in a subset of cases, density of neurons. Similar to previously published procedures [14, 25], five adjacent sections were used to quantify tau inclusions, and three adjacent sections for neurons. For each neocortical section, the crest of the gyrus was traced at 2.5 × from the cortical surface to the white matter, forming a horseshoe shape. The DG was traced by following the outer granule cell layer, and the CA1 was identified and traced using cellular and anatomical landmarks. The top and bottom 1 µm of each section were set as guard height and dimensions of the counting frame were 100 × 100 µm, regardless of magnification. Inclusions were counted if they fell within the z-axis of the chosen dissector height of 3.0 µm. Counting frame rules were modified, where a pathological inclusion was counted if its area was at least 50% in the counting frame, regardless of if it touched the red or green counting frame perimeters. All parameters for analysis were tested and adjusted so that the coefficient of error was < 0.1. For each case, regions of interest were analyzed by a viewer blind to the clinical diagnosis, brain region and brain hemisphere. Regions were analyzed at 63× for Pick bodies and at 40× for neurons. Pick bodies were distinguished based on defined inclusion edges of vesicular appearance, intense AT8 immunohistochemical uptake, and contiguity to a nucleus. About 80 to 200 counting sites were analyzed per neocortical region. Stereological counts obtained were expressed as mean Pick body per cubic millimeter in each region, based on planimetric calculation of volume by the fractionator software.

### Statistical analysis

A Fisher’s exact test was performed to compare ApoE E4 frequency between PPA and bvFTD groups. Welch’s *t*-tests compared mean PMI, education, age at death, age at onset, disease duration, and brain weight between bvFTD and PPA groups. Paired *t*-tests were used to compare overall and regional Pick body densities between hemispheres within clinical groups. Welch’s *t*-tests were used to compare: (1) overall and regional Pick bodies between clinical phenotypes, (2) ratio of overall neocortical to overall hippocampal densities between clinical phenotypes, and (3) overall neocortical and overall hippocampal pathologic densities within clinical phenotypes. Inclusion-to-neuron ratios were calculated (density of inclusions/density of neurons) in a subset of PPA cases, and paired t-tests were utilized to compare inclusion-to-neuron ratios in left MFG and granule cells from the left DG. Statistical analysis was completed using Prism 9 v.9.4.1 (Graphpad). Significance was set at *p* < 0.05.

## Results

### Clinical findings, demographics, and gross patterns of atrophy in PiD cases

The bvFTD and PPA groups did not differ significantly in PMI, education, age at death, age at onset, or disease duration. Mean brain weight of the bvFTD cohort was significantly lower than that of the PPA cohort (*p* < 0.05). There were no statistical differences in ApoE ε4 frequency between clinical groups.

Regions including the hippocampal formation as well as frontal, temporal, parietal, and occipital cortices were examined grossly for atrophy and patterns of hemispheric asymmetry. Five bvFTD cases showed bilateral gross atrophy. Cases 1 & 8 showed rightward asymmetry of atrophy in frontotemporal regions, and Cases 2 & 3 showed leftward asymmetry. Seven PPA cases showed leftward asymmetry of gross cortical atrophy. Case 14, who died early in disease course, showed mild bilateral temporal atrophy. Case 15 showed rightward asymmetry. Atrophy patterns appreciated at gross examination are detailed in Table 2.

**Table 2:** Gross atrophy

Case	Hippocampus	Frontal	Temporal	Parietal	Occipital	Caudate
1	+	+ +	A + , P+ + (R > L)	+	0	0
2	+	L + + + , R + +	+ + + , P STG + +	+	0	+
3	+ + + (L > R)	L + + + , R + +	B + + + (except P STG)	L focally + + + , R + +	L + , R0	B + +
4	+ + +	+ + +	+ + (Poles + + +)	+	+	+
5	+ + +	+ + +	+ +	+ +	+	+
6	+ + +	+ + +	+ + +	+	0	+
7	+ +	+ + +	+	+	0	0
8	+ +	+ + + (R > L)	+ + + (R > L)	+	0	+ +
9	+ +	+ +	+ + +	+	0	+
10	L + + , R + , (A > P)	+ + + (L > R; A > P)	L + + + , R + + (A > P)	L + + , R +	0	+ + +
11	+ + +	+ + + (L > R)	+ + + (L > R)	+ + (L > R)	0	+ + +
12	+ + (L > R)	+ + + (L > R)	+ + + (L > R)	+ (L > R)	0	+ +
13	+ + +	+ + + (knife-edge on L)	L + + + , R + +	L + + + , R + +	0	+ + +
14	+	0	+ + (Poles only)	0	0	0
15	A + + , P +	+	R + , L0	+	0	+
16	+ + (L > R)	+ + + (L > R)	+ + + (L > R)	+ + (L > R)	0	+ +
17	A+ + + , P +	+ +	Poles + + + (L > R)	L + + , R +	+	+
18	+ + +	+ + + (L > R)	+ + + (L > R)	+ + (L > R)	0	+ + +

Semiquantitative grading: 0 absent; + Mild, ++Moderate; +++Severe

*A* Anterior; *P* Posterior; *L* Left; *R* Right; *B* Bilateral; *STG* Superior temporal gyrus

### Distribution and laminar patterns of pick bodies in bvFTD versus PPA

**Fig. 1:**
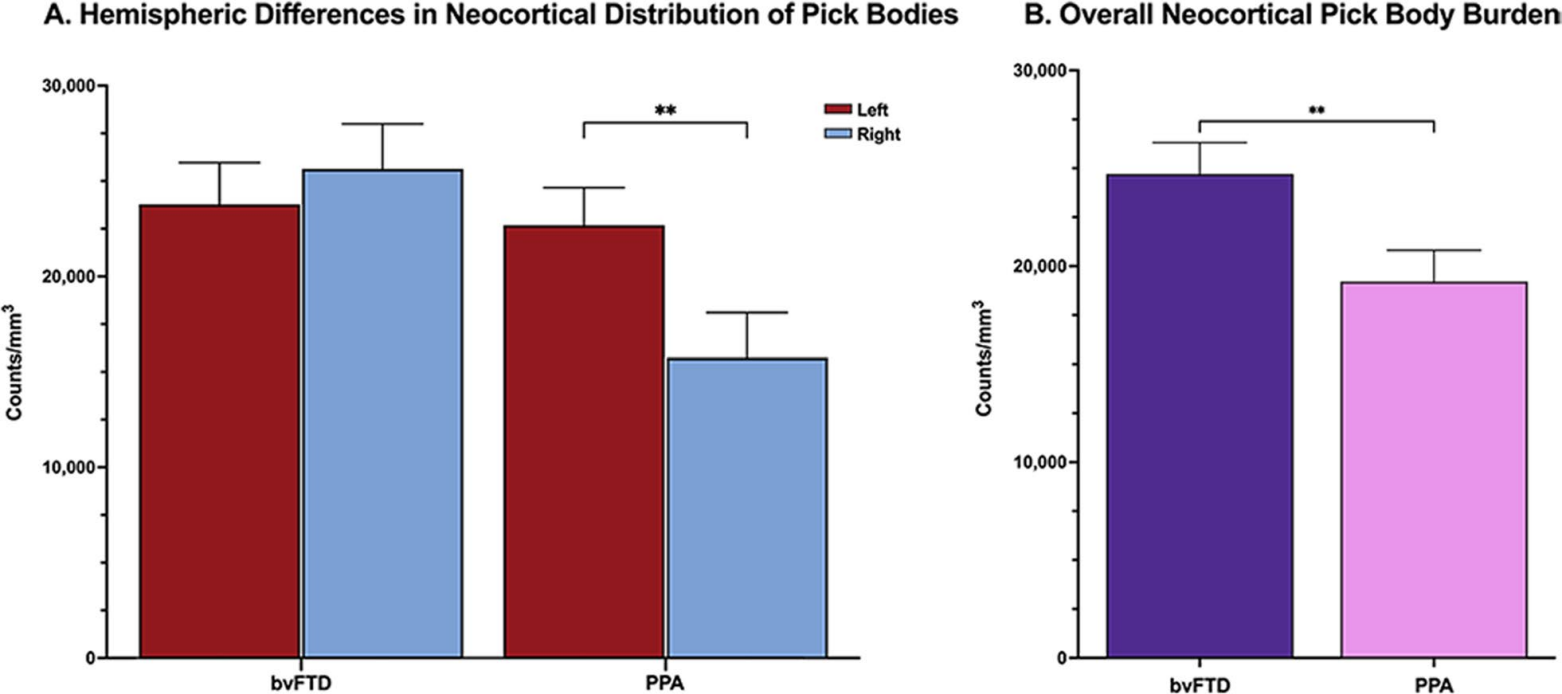
Bilateral and mean total (L + R) neocortical densities of Pick bodies in bvFTD and PPA. Bars represent mean density per cubic millimeter of Pick bodies in neocortical regions in bvFTD (N = 9) and PPA cases (N = 9). Neocortical regions include bilateral middle frontal, superior temporal, and inferior parietal gyri. Error bars represent standard errors of mean (SEM). **A** Bar graphs demonstrate hemispheric differences in mean neocortical density of Pick bodies, highlighting that right hemispheric neocortex in PPA has significantly fewer Pick bodies than the left hemisphere (*p* < 0.01). **B** Bar graphs show the mean overall (L + R) cortical regions in bvFTD vs PPA with error bars representing SEMs. Across the three neocortical regions, bvFTD has significantly greater overall mean density of PiD pathology (M = 25,240.24, SD = 11,431.51) compared to PPA (M = 20,260.76, SD = 11,816.51) (*p* < 0.01), driven by the leftward asymmetry seen in PPA, as shown in **A**. **p < 0.01

Total neocortical neuropathologic densities (MFG + STG + IPL) were combined and compared across hemispheres in bvFTD and PPA. In bvFTD, combined neocortical neuropathologic burden was bilateral (Fig. 1A). In PPA, combined densities in the left hemisphere were similar to left and right hemispheric densities in bvFTD; the right hemisphere in PPA was significantly less affected by PiD compared to the language-dominant left hemisphere (*p* < 0.01). Overall (left + right MFG, STG, IPL) neocortical neuropathologic burden was significantly greater in bvFTD than PPA (*p* < 0.01) (Fig. 1B). Highest neocortical densities of Pick bodies were found in ATL (M = 31,634, SD = 10,502) in PPA, whereas peak densities were evident in the MFG in bvFTD (M = 26,036, SD = 8719). There were higher inclusion densities in the right STG in bvFTD (M = 27,537.25, SD = 17,094.79) compared to right STG in PPA (M = 8123.19, SD = 6870.01), and in the right MFG in bvFTD (M = 29,157.41, SD = 7,827.25) compared to right MFG in PPA (M = 20,176.16, SD = 12,193.21); the former reached significance (*p* < 0.01; *p* = 0.08, respectively).

Case 11 was the sole case with a clinical diagnosis of the semantic variant of PPA. Distribution of Pick bodies closely followed PPA cohort trends, where bilateral ATL was the most affected neocortical region (M = 29,783.86), and bilateral DG was the region with the most abundant Pick bodies (M = 56,738.8). MFG was the next most affected neocortical region in this case, with the left hemisphere more affected than right (left MFG = 28,720.86 inclusions per mm^3^; right MFG = 20,598.53 inclusions per mm^3^).

**Fig. 2:**
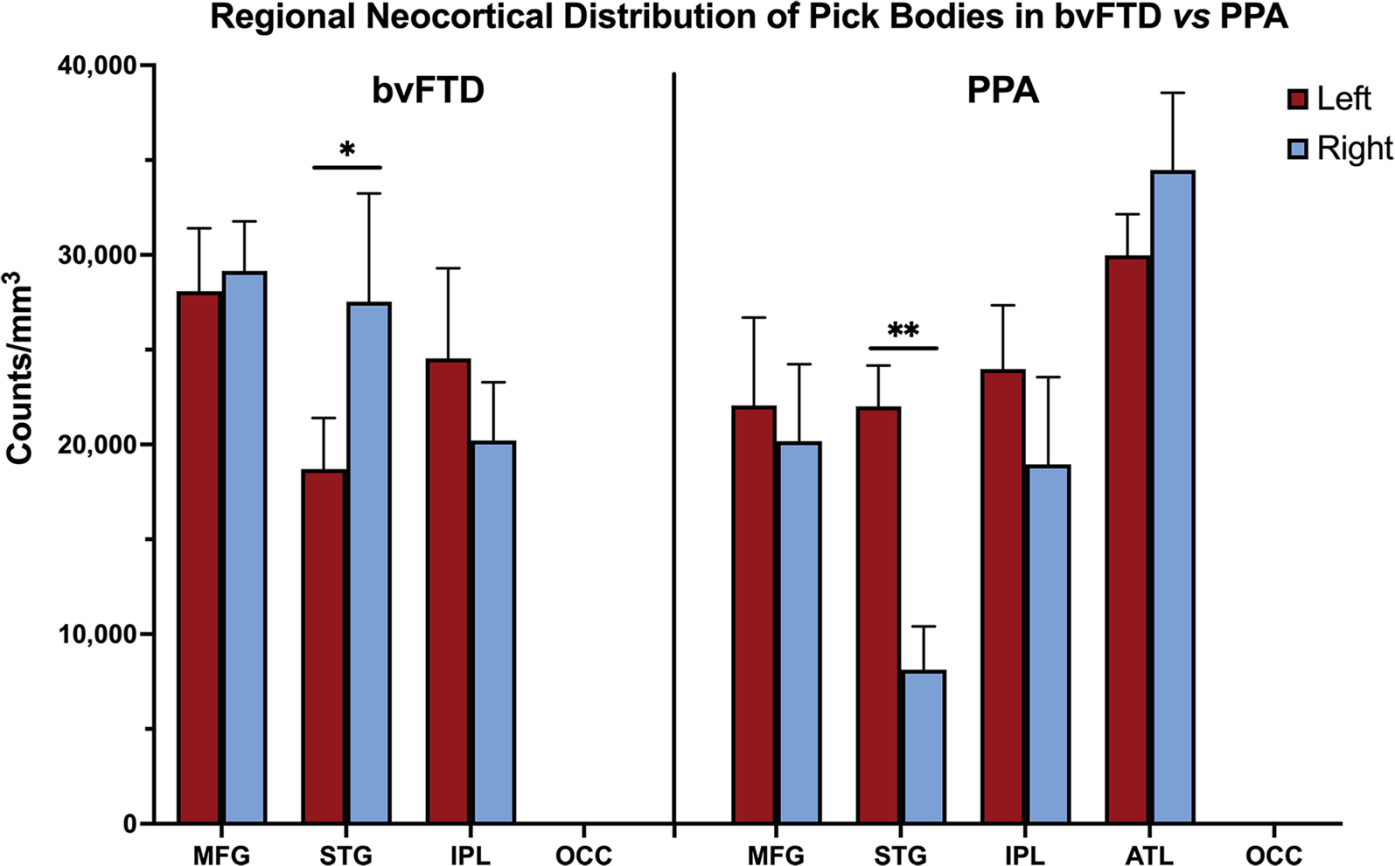
Neocortical distribution of Pick bodies in bvFTD vs PPA. Height of bars represent mean density per cubic millimeter of Pick bodies in neocortical regions in bvFTD (N = 9) and PPA (N = 9) cases. Error bars represent standard errors of mean (SEM). Pick body density was highest in MFG in bvFTD and ATL in PPA, and OCC showed no Pick bodies in bvFTD and PPA. bvFTD displayed generally bilateral distribution of PiD pathology, except for STG which showed rightward asymmetry (*p* < 0.05). In PPA, regions were generally leftward asymmetric, which reached significance in STG (*p* < 0.01). ATL showed slight rightward predilection in PPA. MFG = Middle frontal gyrus; STG = Superior temporal gyrus; IPL = Inferior parietal lobule; ATL = Anterior temporal lobe; OCC = Occipital cortex. *p < 0.05; **p < 0.01

In all cases, as expected, the occipital cortex showed extremely sparse to no pathology in either group. See Fig. 2 for all neocortical stereologic densities.

Pick body distribution across laminar layers was difficult to discern given immense neurodegeneration and resulting spongiotic tissue. Qualitative analysis by a neuropathologist (RJC) noted Pick body inclusions present in superficial layers II and III and deep layers V and VI. Relative absence of Pick bodies in layer IV was a unifying laminar feature across phenotypes. In regions with lower PiD densities, superficial layers were more populated with Pick bodies, with layer II mildly more affected (Additional file 1: Fig. S1). In more affected regions, the layer II was devastated with severe statis spongiosis.

### Hemispheric asymmetry of neocortical Pick pathology in PPA compared to bvFTD

**Fig. 3:**
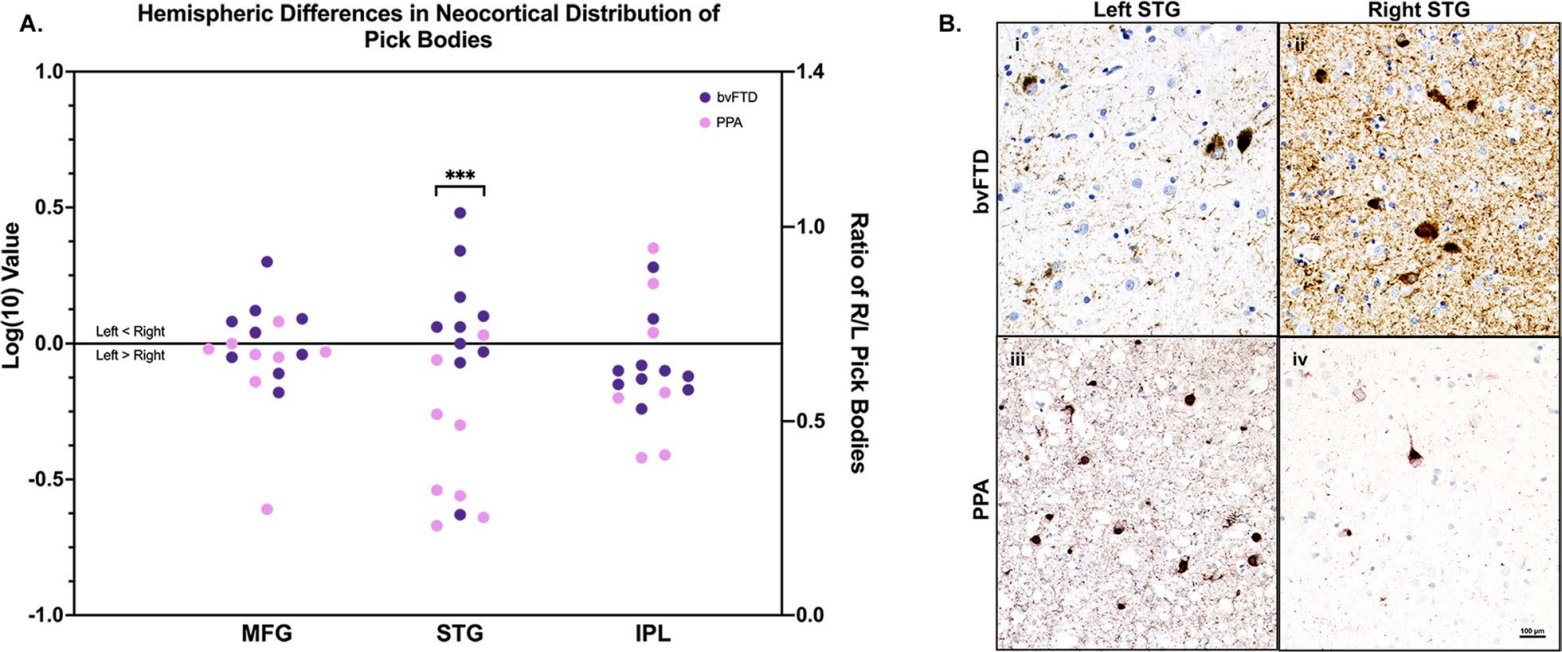
Laterality of Pick bodies in Right/Left Neocortex. **A** Ratios of Pick body density counts per millimeter cubed in individual right/left neocortical regions were transformed logarithmically (base 10) to illustrate hemispheric differences in PiD pathology in bvFTD (N = 9) and PPA (N = 9). In STG, right-to-left ratio was significantly different between PPA, which showed significant leftward asymmetry, and bvFTD, which showed slight rightward asymmetry (*p* < 0.001). The IPL ratio for Case 14 was excluded because the right IPL showed no pathology, so the right/left ratio equaled zero and thus could not be logarithmically transformed. The MFG ratio for Case 15 was excluded as an outlier (M ±  ≥ 2 SD). MFG = Middle frontal gyrus; STG = Superior temporal gyrus; IPL = inferior parietal lobule; R = Right; L = Left. ***p < 0.001. **B** Opposite laterality of Pick bodies in STG of PPA vs bvFTD. Photomicrographs **(i)** & **(ii)** were obtained from Case 5, an 82-year-old female with a 12-year history of bvFTD and **(iii)** and **(iv)** were obtained from Case 12, a 65-year-old male with a 12-year history of PPA-G; images highlight significant leftward asymmetry in PiD in STG, while bvFTD cases showed slight rightward predominance. Brown appearance in **i** and **ii** are due to DAB, and red appearance in **(iii)** and **(iv)** are due to Vector NovaRed substrate. Scale bar = 100 µm in **(iv)**, and also applies to **(i–iii)**

Concordant with the aphasic phenotype, left neocortical areas in PPA had significantly greater pathologic burden than right (*p* < 0.05) (see Fig. 1A). In PPA, the STG showed significant asymmetric pathologic burden (L > R, *p* < 0.01) (Fig. 2). IPL and MFG was also leftward asymmetric in PPA, though this did not reach significance. Interestingly, the ATL showed slight rightward predominance. Neocortical distributions of Pick bodies in bvFTD were generally symmetric except for the STG, which showed significant rightward asymmetry (*p* < 0.05). The ratio of right to left densities were also calculated for neocortical regions in both groups, where ratios > 1 indicated more pathology in the right hemisphere, and ratios < 1 indicated greater leftward pathology. Ratios were then transformed logarithmically, where values > 0 indicated more rightward pathology and values < 0 indicated more leftward pathology. Figure 3 shows logarithmically transformed ratio values for each case. According to logarithmically transformed ratios, the direction of asymmetry in STG was significantly different between PPA and bvFTD (*p* < 0.001); in PPA, STG pathology was leftward predominant (M = -0.39, SD = 0.28), while in bvFTD cases it was more rightward (M = 0.14, SD = 0.19). In PPA, MFG was slightly leftward-predominant (M = -0.10, SD = 0.22) while MFG was generally bilateral in bvFTD (M = 0.03, SD = 0.14). In IPL, both bvFTD (M = − 0.05, SD = 0.14) and PPA (M = − 0.1, SD = 0.28) cases generally showed leftward predominance. The significant difference in STG between the two clinical groups was detectable through qualitative microscopical examination (Fig. 3B).

### Symmetric hippocampal predominance of Pick pathology regardless of clinical phenotype

**Fig. 4:**
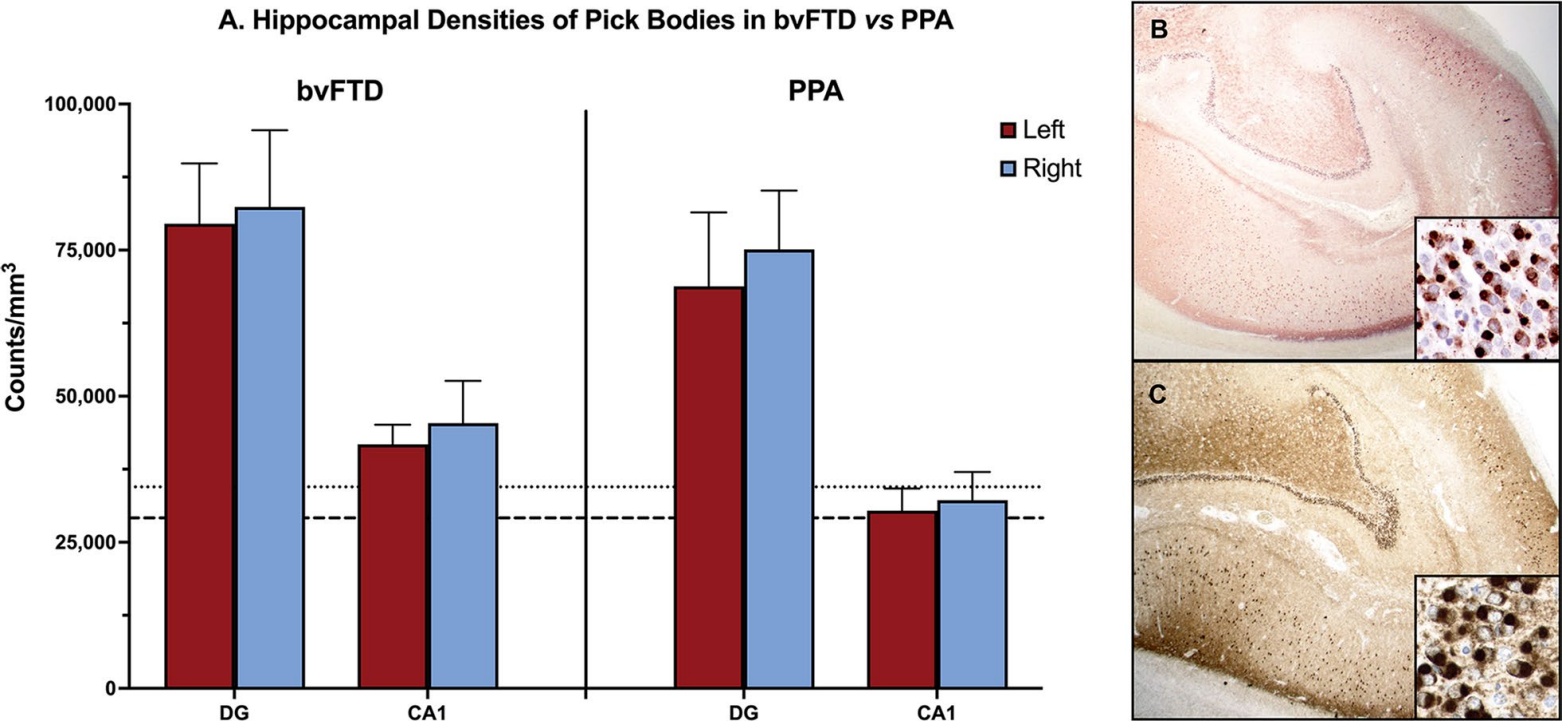
Abundant hippocampal pathologic burden is a universal substrate of Pick’s Disease (PiD). **A** Height of bars represent mean density per cubic millimeter of Pick bodies in anatomic regions in bvFTD (N = 9) and PPA cases (N = 9). Error bars represent standard errors of mean (SEM). Dotted line indicates the highest mean neocortical Pick body density from Fig. 2 for the PPA group, which was right ATL at 34,473 inclusions per mm^3^. Dashed line indicates the highest mean neocortical Pick body density from Fig. 2 for the bvFTD group, which was right MFG at 29,157 inclusions per mm^3^. bvFTD cases had slightly greater mean DG and CA1 densities compared to PPA cases, which did not reach significance. Burden was symmetric for both phenotypes. Photomicrographs (**B** & **C**) illustrate the substantial neuropathologic burden throughout the entire hippocampus in both the behavioral and aphasic phenotypes; **B** was obtained from Case 5, an 82-year-old female with a 12-year history of bvFTD and **C** was obtained from Case 12, a 65-year-old male with a 12-year history of PPA-G. Inset highlights the granule cells of the dentate gyrus, which were most affected by Pick bodies in both bvFTD and PPA cohorts. Images were taken at 1 × magnification, and insets were taken at 20X. DG = Dentate gyrus; CA1 = Cornu Ammonis field 1

In both bvFTD and PPA cases, a very high density of pathology was found in the dentate gyrus (DG) and CA1 regions of the hippocampus. No hemispheric differences in DG and CA1 densities were found (Fig. 4A). In both the bvFTD and PPA groups, significantly greater pathology was measured in the hippocampus (DG + CA1) than the entire neocortex combined (MFG + STG + IPL) (*p* < 0.0001). There was no significant difference in ratio of neocortical to hippocampal burden between phenotypes (Fig. 4B and C). Even Case 14, who died at an early disease stage, had relatively high density of Pick bodies present in the DG (~ 2000 inclusions per mm^3^).

**Fig. 5:**
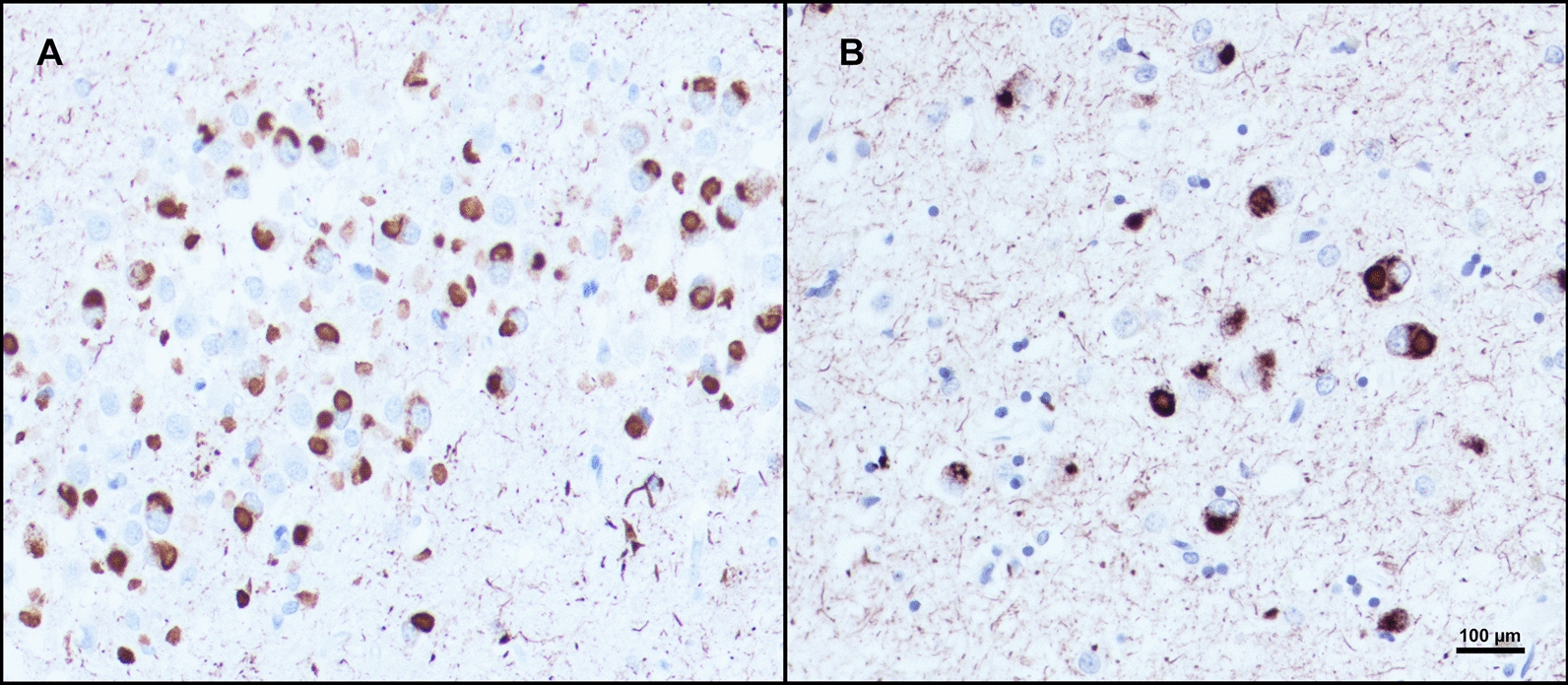
Pick body packing density in hippocampus versus neocortex. **A** and **B** show density of tau pathology staining using immunohistochemistry with AT8 antibody in relation to neuronal density. **A** illustrates the high degree of pathology in the dentate gyrus (DG) of the hippocampus, which also holds a high density of neurons, in Case 13, a 75-year-old male with a 9-year history of PPA. **B** shows relative density of tau pathology in the left middle frontal gyrus (MFG), a neocortical region with relatively abundant Pick disease pathology, in Case 18, a 71-year-old female with a 14-year history of PPA. Photographs were taken at the same magnification. Inclusion-to-neuron analyses in a subset of cases show that despite differences in neuronal packing density, hippocampal cells, particularly granule cells in the DG, contain significantly more inclusions per neuron (*p* < 0.05). Bar indicates 100 µm

In a subset of PPA cases (Cases 10, 16, and 17), neurons were quantified in the left MFG, and an inclusion-to-neuron ratio was calculated to estimate the relationship between presence of inclusions and neuronal packing density (Fig. 5). Left MFG was quantified due to severe pathologic burden with relatively less severe neuronal loss (atrophy). Inclusion-to-neuron ratio for the granule cells of the left DG in the same cases was previously collected in Kawles et al. [25]. On average, 9.8% of neurons in left MFG contained a Pick body. In contrast, about 66.9% of granule cells in these three cases contained an inclusion. This difference was statistically significant (*p* < 0.05) (Additional file 1: Fig. S2).

## Discussion

Until recently, Pick’s disease was considered a relatively uniform clinicopathologic entity that described a frontotemporal lobar degeneration with pathologic 3R-tau-positive intraneuronal inclusions known as Pick bodies. In actuality, the pathologic diagnosis of PiD can be found in two separate, distinct clinical phenotypes: the aphasic dementia of PPA and the comportmental dementia of bvFTD. The present study aimed to distinguish regional and hemispheric distributions of Pick bodies in a well-characterized cohort of PPA- and bvFTD-PiD individuals. Pick bodies showed a leftward predilection in the neocortex in PPA cases concordant with the aphasic phenotype, while bvFTD cases were on average bilaterally affected. Differential vulnerability of the hemispheres in PPA versus bvFTD was particularly pronounced in the STG, where pathology was significantly left-predominant in PPA and right-predominant in bvFTD. Lastly, the hippocampus—primarily the dentate subregion—was uniquely and universally affected with a high burden of Pick bodies in all cases regardless of clinical phenotype.

The left-lateralization of atrophy in PPA is a core biologic feature of the aphasic phenotype [15, 34, 38, 41]. Asymmetric pathologic inclusion distribution has been shown in PPA associated with TDP-43 [26, 27] and in PPA associated with ADNC [14]. A seminal study found that distribution of AD pathology differed between PPA and dementia of the Alzheimer’s type, where PPA due to AD showed pronounced leftward asymmetry of AD neurofibrillary tangle pathology compared to symmetric distribution in the amnestic phenotype. This study was one of the first to demonstrate clinicopathologic concordance between distribution of the same pathologic entity in different clinical syndromes [14]. The current study is also the first to demonstrate this concordance in PPA due an FTLD-tauopathy using stereologic analysis. Our findings further showed symmetric overall neocortical predominance of Pick bodies in bvFTD, a finding that has been well-documented in both imaging and histologic analyses [22, 47, 60]. Overall left neocortical Pick body densities in PPA are comparable to the overall left and right hemisphere Pick body densities in bvFTD, consistent with previous evidence of PPA as a disease of the language dominant hemisphere and bvFTD as a bilateral disease. Surprisingly, findings reveal that the observed asymmetry in PPA-PiD is not characterized by increased left hemisphere pathology, but rather decreased right hemispheric pathology compared to bvFTD-PiD. Such results provide insight into the progression of PiD in these phenotypes, where the right hemisphere appears relatively spared in PPA-PiD until later disease stages, while PiD underlying bvFTD may engender and spread bilaterally.

The STG emerged as an interesting region of differential vulnerability, whereby PPA cases showed significant left-predominant pathology while bvFTD patients showed significant right-sided predominance. The left STG is a well-established component of the human language network, though its exact function is still debated. The left STG is classically associated with Wernicke’s area, which was first characterized as a site of language comprehension based on lesion anatomy in stroke patients [50, 59]. However, neurodegenerative research reveals that comprehension deficits can arise from a constellation of cortical atrophy sites in addition to the classic Wernicke’s area/left STG [2, 10, 40, 46]. Several PPA studies have instead found the anterior and posterior segments of the left STG to underly separate and distinct functions that correlate with different language deficits [39, 61]. The anterior temporal lobe, which includes the anterior STG, is found to assist with functions such as word comprehension and object naming, the proposed functions of Wernicke’s area [9, 19, 36, 42]. Conversely, the posterior STG supports the phonological loop, which aids in word repetition, sentence comprehension, and the storage of auditory speech input in working memory for future articulation [17, 39, 55]. The agrammatic phenotype more closely aligns with posterior STG impairment, which correlates with our pathologic findings. It is important to note that while a minority of cases show moderate to frequent neuritic plaques, this pathologic entity likely does not contribute to the aphasic or behavioral phenotype [14]. Though the functional modularity of the right temporal lobe is less understood, it is generally responsible for auditory and emotional processing [5, 43, 63]. In FTD cohorts, right-predominant temporal lobe atrophy is most often associated with severe comportment and personality changes [23, 56]. Right-asymmetric temporal PiD pathology in bvFTD is therefore concordant with observed behavioral symptoms. However, bvFTD-PiD shows peak atrophy patterns in bilateral prefrontal and anterior temporal cortices; therefore, PiD pathology in right STG may serve as a neighborhood marker for more severe burden in anterior regions.

Despite relative sparing of memory functioning in the behavioral and aphasic phenotypes, we found the hippocampus to be greatly affected in both bvFTD- and PPA-PiD. The selective vulnerability of the granule cells of the dentate gyrus in PiD has previously been reported [11, 12, 21, 25]. A recent study by Mesulam et al. (2021) demonstrated that PPA patients with underlying AD neuropathology show preserved memory functioning despite hippocampo-entorhinal postmortem neuropathologic burden that is comparable to those with amnestic dementia due to AD [37]. The finding that DG and CA1 pathology is significantly greater than overall neocortical pathology in both diseases is striking. In a recent study, our group found that, on average, about 60% of dentate granule cells contained a Pick body [25]. Yet despite intense hippocampal PiD pathologic burden, PPA and bvFTD patients show relative sparing of memory functioning. One variable that was thought to account for increased Pick body density in the hippocampus is higher packing density of granule cells in the DG and, to a lesser extent, pyramidal cells in the CA1, compared with the packing density of neocortical neurons. However, when we compared the inclusion-to-neuron ratios of granule cells to neurons in the left MFG in three PPA cases, we found that DG granule cells *still* showed significantly greater burden of PiD. Further, the average inclusion-to-neuron ratio from the three chosen cases (66.9%) aligns with previous published findings of 60% [25]. Hippocampal cells thus appear to be resilient to potential deleterious effects of Pick-related tau accumulation. One possible explanation for this resilience is that while the Pick body may form early in disease course, their presence may not necessarily lead to neurodegeneration; indeed, our group has shown relative preservation of granule cells in the DG in both PiD and TDP-43 proteinopathies, despite pathologic accumulation [25, 26]. Another explanation is that despite early arrival to the dentate gyrus, PiD may progress at a slower rate in the hippocampus compared to neocortex, leading to preserved memory until later disease stages. Nevertheless, we found the DG showed at least 3-times more Pick bodies than neocortical regions in both bvFTD and PPA. Results suggest the hippocampus is vulnerable to Pick body pathologic accumulation, regardless of phenotype, but this vulnerability is not necessarily associated with expected impairments in memory domains.

Despite a unique clinical presentation of semantic impairments, Case 11 followed similar patterns of pathologic distribution compared to the other PPA cases with agrammatic presentations. There was one notable exception in regional vulnerability; the semantic PPA case was more heavily affected by Pick body pathology in the MFG whereas the agrammatic cases showed greater STG involvement. While differences between PiD causing agrammatic versus semantic deficits cannot be extrapolated from one case, comprehensive analyses can offer clues regarding relative spread and associated cognitive dysfunction.

The present study contains one of the largest cohorts of autopsy confirmed PiD leading to two disparate clinical phenotypes. Stereological analysis provided rigorous quantitative measures of inclusion density that allow for reliable comparison of pathologic density between a large host of regions. Limitations include small sample size and lack of ATL data in the bvFTD group and neuronal density data from all cases. Given severe degeneration of tissue, neuronal density data would illuminate whether lower pathologic inclusion density correlates with lower neuronal number, implying inclusions are cleared as neurons are lost [26]. Lastly, our modified stereologic methodology analyzes adjacent 5 µm sections; this methodological approach may result in overestimation of inclusion densities. Subsequent analyses will include stereological quantification of subcortical regions in PiD as well as collection of similar data in 4R-FTLD-tauopathies, corticobasal degeneration and progressive supranuclear palsy, to determine shared versus unique neuropathologic signatures of 3R and 4R tauopathies. Additional analyses are also needed to understand the contributions of neuroinflammatory, synaptic, and genomic hallmarks that may lead to distinct clinical phenotypes within the same pathology. Together, these studies may determine the individual features that create the complex clinicopathologic picture of frontotemporal dementias.

### Supplementary Information

**Additional file 1: Fig. S1.** Laminar distribution in bilateral superior temporal gyri (STG). **Fig. S2.** Inclusion-to-neuron ratio in a subset of PPA-PiD cases.

## Data Availability

The datasets used and/or analyzed during the current study are available from the corresponding author on reasonable request.

## Abbreviations

PiD: Pick’s disease
FTLD: Frontotemporal lobar degeneration
PPA: Primary progressive aphasia
bvFTD: Behavioral variant frontotemporal dementia
MFG: Middle frontal gyrus
STG: Superior temporal gyrus
IPL: Inferior parietal lobule
ATL: Anterior temporal lobe
DG: Dentate gyrus
OCC: Occipital cortex
ADNC: Alzheimer’s disease neuropathologic change
