# Supplementary material for: Phenotypically concordant distribution of pick bodies in aphasic versus behavioral dementias
Source: Acta Neuropathol Commun. 2024 Feb 22;12:31. doi: 10.1186/s40478-024-01738-7 (PMC10885488; doi:10.1186/s40478-024-01738-7)
Supplement: Supplementary file 1 — Additional file 1: Fig. S1. Laminar distribution in bilateral superior temporal gyri (STG). Fig. S2. Inclusion-to-neuron ratio in a subset of PPA-PiD cases. [file 40478_2024_1738_MOESM1_ESM.docx]

**
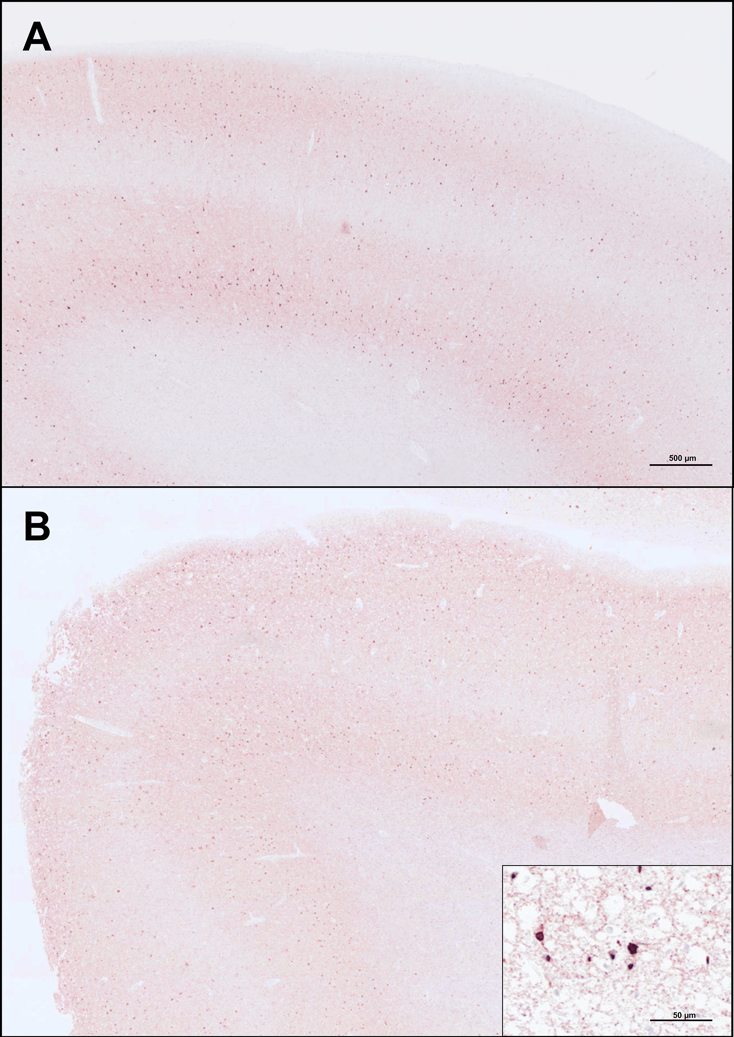
**

**Fig. S1. Laminar distribution in bilateral superior temporal gyri (STG). (A-B)** show distribution of tau pathology staining using immunohistochemistry with AT8 antibody. (**A**) depicts right superior temporal gyrus (STG) in Case 8, a 61-year-old male with a 14-year history of bvFTD, and (**B**) depicts left STG in Case 12, a 65-year-old female with a 12-year history of PPA. Photographs were taken at 1x magnification. Inclusions can be appreciated in both superficial and deep cortical layers, with relative sparing of layer IV neurons. The superficial layers are particularly atrophic; inset depicts an example of spongiotic tissue seen at layer II.


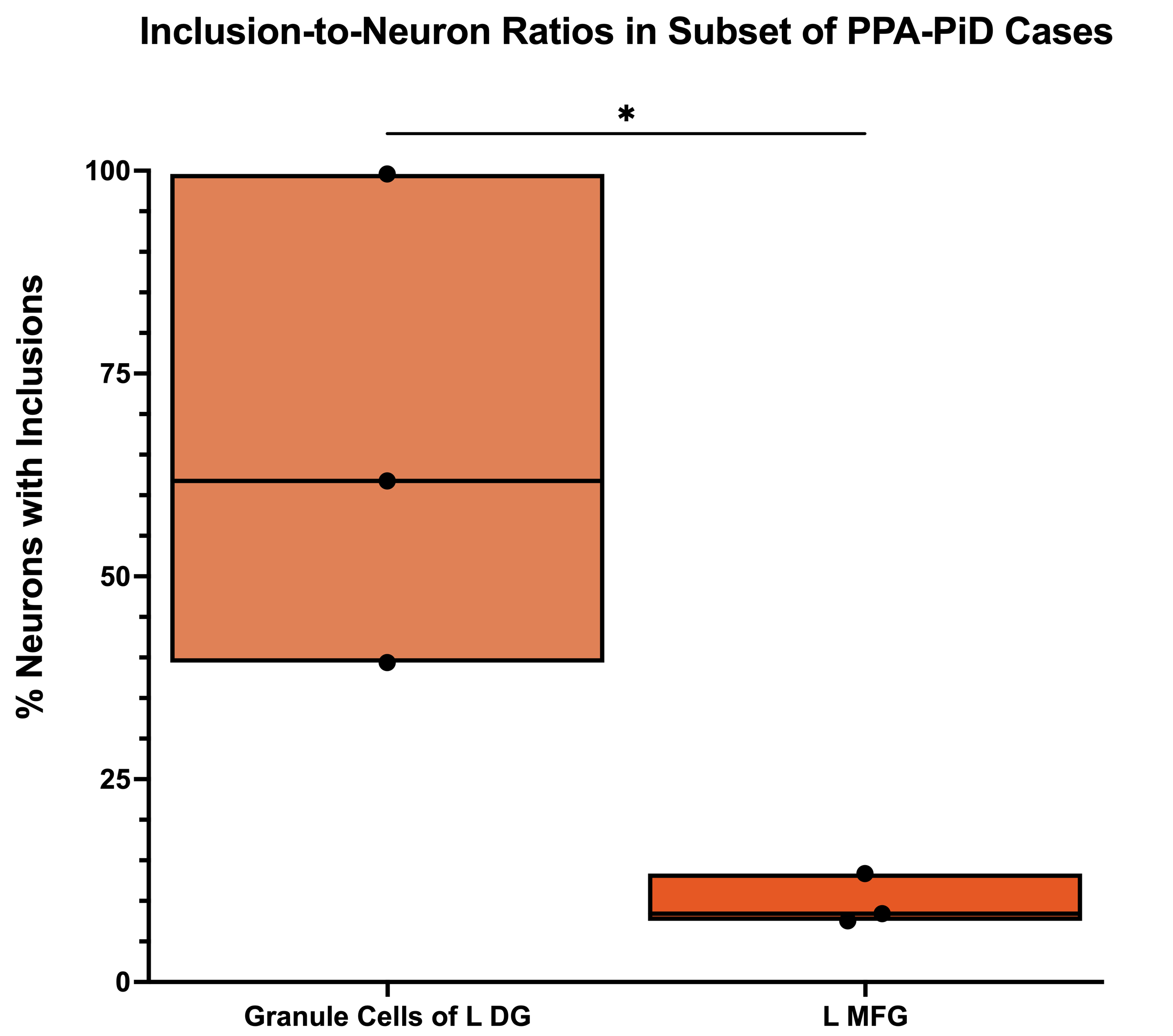


**Fig. S2. Inclusion-to-neuron ratio in a subset of PPA-PiD cases.** Height of bar represent percent of neurons containing an inclusion in the left dentate gyrus (DG) and left middle frontal gyrus (MFG) in a subset of PiD cases (N=3) with a clinical diagnosis of PPA. In DG, ratio was calculated for granule cell layer only. Ratios indicate that despite greater neuronal packing density in the DG, a significantly higher percentage of neurons are affected (i.e., contain an inclusion) compared to a neocortical like the MFG. The box delineates minimum and maximum densities, and the line within the box represents median. Each point represents an individual case. **p*<0.05
